# Supplementary figures and images for: The genotype distribution, infection stage and drug resistance mutation profile of human immunodeficiency virus-1 among the infected blood donors from five Chinese blood centers, 2014–2017
Source: PLoS One. 2020 Dec 21;15(12):e0243650. doi: 10.1371/journal.pone.0243650 (PMC7752150; doi:10.1371/journal.pone.0243650)

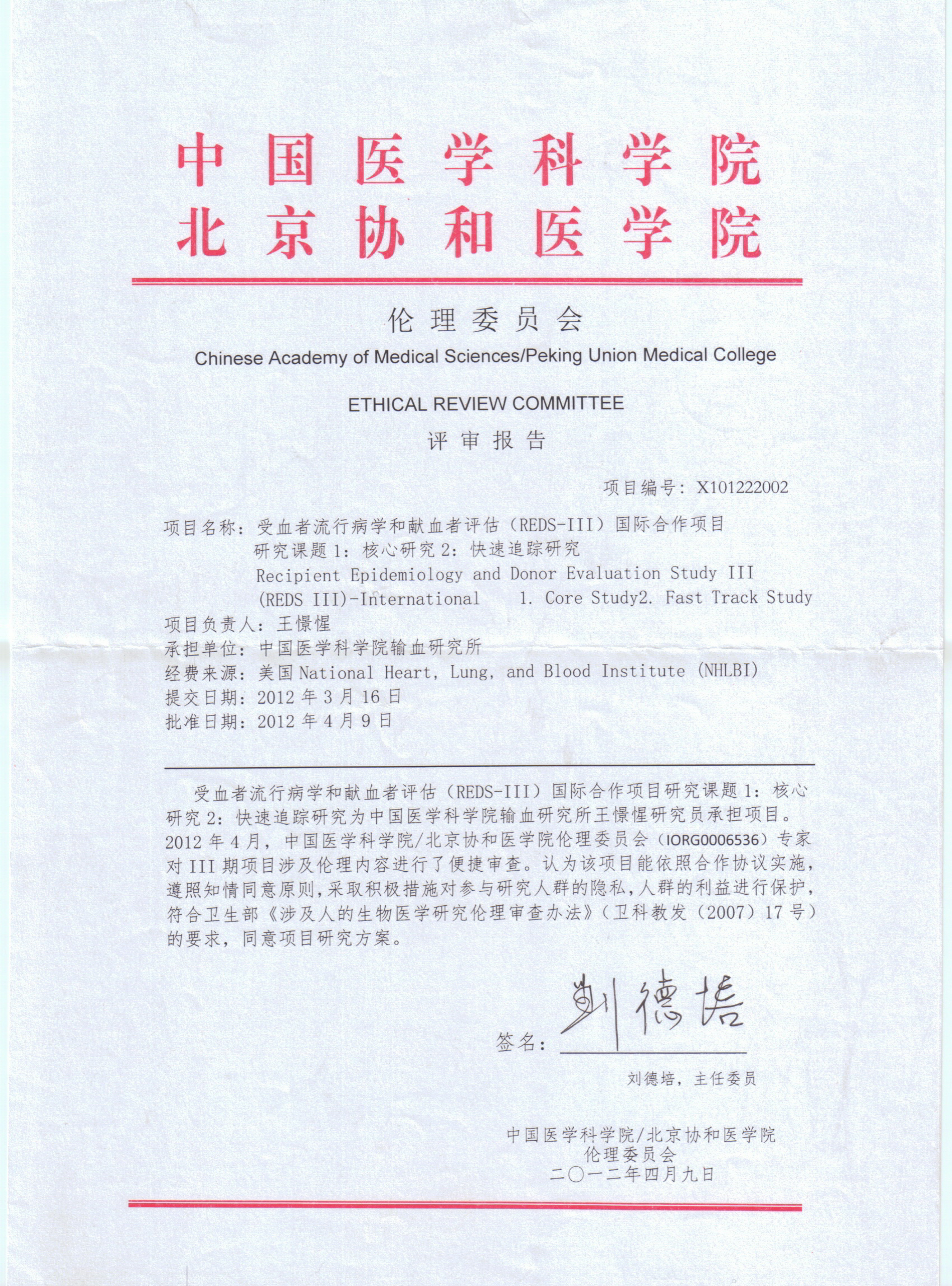

Supplement: S1 Fig — (TIF) [file pone.0243650.s001.tif]

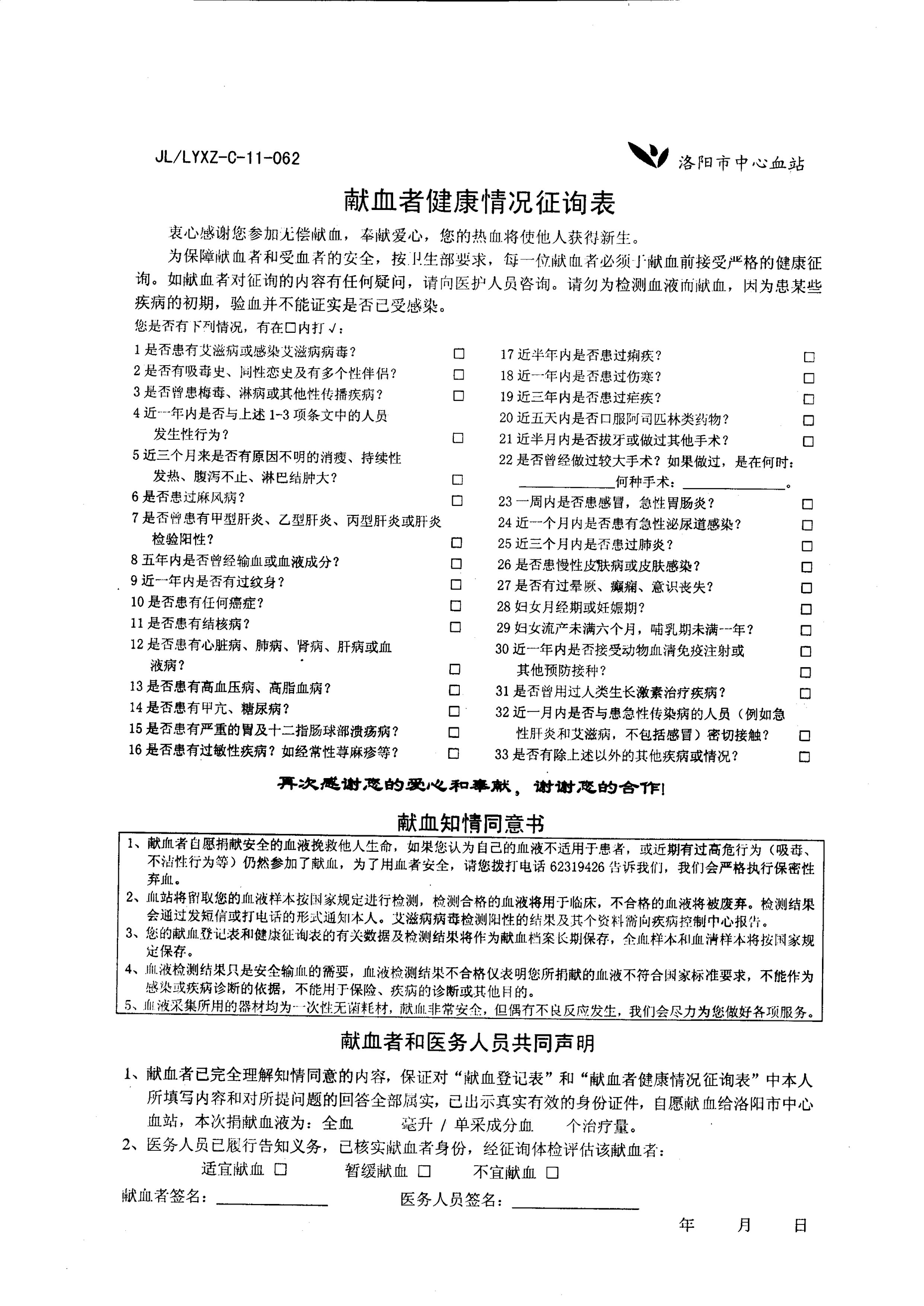

Supplement: S2 Fig — (TIF) [file pone.0243650.s002.tif]
