# Supplementary material for: The genotype distribution, infection stage and drug resistance mutation profile of human immunodeficiency virus-1 among the infected blood donors from five Chinese blood centers, 2014–2017
Source: PLoS One. 2020 Dec 21;15(12):e0243650. doi: 10.1371/journal.pone.0243650 (PMC7752150; doi:10.1371/journal.pone.0243650)
Supplement: S1 Table — (DOC) [file pone.0243650.s003.doc]

Supporting information

S1Table .HIV-1 genotypic drug resistance mutations

| Specimen ID | Infection stages | Genotyping | PI accessory DRMs (n = 7) | PI major DRMs (n = 2) | NRTI DRMs (n = 7) | NNRTI DRMs (n = 32) | Drug resistance |
| --- | --- | --- | --- | --- | --- | --- | --- |
| CQ15004978 | LONG-TERM | 07_BC |  |  | D67N |  | LLR to ABC AZT TDF |
| CQ15004954 | LONG-TERM | 55_01B |  |  |  | V179E | PLR to EFV,ETR NVP,RPV |
| CQ15004939 | LONG-TERM | 08_BC |  |  |  | V179E | PLR to EFV ETR NVP,RPV |
| CQ15004936 | LONG-TERM | 07_BC | Q58E |  |  |  | PFR to NFV,LLR to TPV |
| CQ15004799 | LONG-TERM | 07_BC | Q58E |  |  |  | PLR to NFV,LLR to TPV |
| CQ14001771 | LONG-TERM | 07_BC |  | M46L |  |  | PLR to ATV LPV |
| CQ14001319 | LONG-TERM |  |  |  | D67N | K103N | LLR to ABC AZT TDF PLR to FTC 3TC |
| CQ14000684 | LONG-TERM | 08_BC |  |  |  | E138A/  Y188D | PLR to ETR LLR to RPV |
| CQ14000658 | LONG-TERM | 01_AE |  |  | M184L | V179E | PLR to EFV ETR NVP,RPV |
| CQ13003228 | LONG-TERM | 07_BC | Q58E |  |  |  | LLR to TPV |
| CQ13003037 | LONG-TERM | 55_01B |  |  |  | V179E | PLLR to EFV, ETR RPV and NVP |
| CQ13002852 | LONG-TERM | 07_BC |  |  |  | K238N | PLLR to EFV and NVP |
| CQ13002447 | LONG-TERM | 07_BC |  |  |  | V179D | PLLR to EFV, ETR  RPV and NVP |
| CQ13001603 | LONG-TERM | 07_BC |  |  |  | V179D | PLLR to EFV, ETR RPV and NVP |
| CQ13001278 | LONG-TERM | 07_BC |  |  | K70Q |  | PLLR to 3TC, ABC D4T, DDI, FTC and TDF |
| CQ13001022 | LONG-TERM | 08_BC |  |  |  | E138A | PLLR to ETR; LLR to RPV |
| CQ13000959 | RECENT | 08_BC |  |  |  | E138A | PLLR to ETR; LLR to RPV |
| cq12004551 | RECENT | 08_BC |  |  |  | E138A | PLLR to ETR; LLR to RPV |
| cq12004326 | LONG-TERM | 07_BC |  |  |  | V179D | PLLR to EFV, ETR  RPV and NVP |
| cq12004297 | LONG-TERM | 08_BC |  |  |  | V179D | PLLR to EFV, ETR RPV and NVP |
| cq12004073 | RECENT | 55_01B |  |  |  | V179E | PLLR to EFV, ETR RPV and NVP |
| CQ12003834 | LONG-TERM | 07_BC |  |  |  | V179D | PLLR to EFV, ETR RPV and NVP |
| CQ12003677 | LONG-TERM | 07_BC |  |  | K219R | K238N | PLLR to AZT, D4T, EFV and NVP |
| CQ12003214 | LONG-TERM | 07_BC |  | M46I |  |  | PLLR to ATV, FPV, IDV and LPV; LLR to NFV |
| CQ12002493 | LONG-TERM | 08_BC |  |  |  | V179E | PLLR to EFV, ETR RPV and NVP |
| CQ12000196 | LONG-TERM | 07_BC | L23I |  |  |  | LLR to NFV |
| MY14001617 | RECENT | 55_01B |  |  |  | V179E | PLLR to EFV ETR NVP RPV |
| MY14001231 | RECENT | 01_AE |  |  |  | K103N | HLR to EFV NVP |
| MY14000058 | LONG-TERM | 07_BC | Q58E |  |  |  | PFR to NFV,LLR to TPV |
| LY14003600 | LONG-TERM | 55_01B |  |  |  | V179E | PLLR to EFV,ETR RPV and NVP |
| LY12004233 | RECENT | 07_BC |  |  |  | E138G, G190E | HLR to EFV,NVP and RPV IR to ETR |
| LY12003986 | LONG-TERM | 55_01B |  |  |  | V179E | PLLR to EFV,ETR RPV and NVP |
| GX14002550 | LONG-TERM | 07_BC | I84M |  |  |  |  |
| GX14001902 | LONG-TERM | 59_01B |  |  | D67N |  | LLT to ABC AZT TDF |
| WS17000127 | LONG-TERM | 07_BC | Q58E |  |  |  | PLLR to NFV LLR to TPV |
| WS15001148 | LONG-TERM | 07_BC |  |  |  | A98G | LLR to DOR ,EFV ETR RPV PLR to ETR  IR to NVP |
| WS15000652 | LONG-TERM | 07_BC |  |  | D67N |  | LLR to AZT |
| WS15000162 | LONG-TERM | 55_01B |  |  |  | V179E | PLR to EFV ETR NVP RPV |
| WS13003163 | LONG-TERM | 07_BC |  |  |  | V179D | PLLR to EFV, ETR, RPV and NVP |
| WS12001503 | LONG-TERM | 55_01B |  |  |  | V179E | PLLR to EFV, ETR, RPV and NVP |
| ws12001012 | LONG-TERM | 01_AE |  |  |  | V179D | PLLR to EFV, ETR, RPV and NVP |
| WS12000352 | LONG-TERM | 07_BC |  |  |  | V179D | PLLR to EFV, ETR, RPV and NVP |
| WS12000312 | LONG-TERM | 55_01B |  |  |  | V179E | PLLR to EFV, ETR, RPV and NVP |

(a) As determined by the Stanford HIVdb Program Genotypic Resistance Interpretation Algorithm (http://hivdb.stanford.edu). Potential Low level Resistance (PLLR), Low Level Resistance (LLR), intermediate level resistance (ILR) or high-level resistance (HLR); NFV, Nelfinavir; EFV, efavirenz; ETR, etravirine; RPV, rilpivirine; NVP, nevirapine; ATV, atazanavir; FPV, Fosamprenavir; IDV, indinavir; LPV, lopinavir; AZT, Zidovudine; D4T, Stavudine; 3TC, Lamivudine; ABC, Abacavir; D4T, Sanilvudin; DDI, didanosine; FTC, Emtricitabine; TDF, Tenofovir disoproxil fumarate; TPV, tipranavir; SQV, saquinavir.
