# Supplementary material for: The genotype distribution, infection stage and drug resistance mutation profile of human immunodeficiency virus-1 among the infected blood donors from five Chinese blood centers, 2014–2017
Source: PLoS One. 2020 Dec 21;15(12):e0243650. doi: 10.1371/journal.pone.0243650 (PMC7752150; doi:10.1371/journal.pone.0243650)
Supplement: Attachment — Submitted filename: Response to Reviewers.docx [file pone.0243650.s009.docx]

Dear Editors and Reviewers:

Thank you for your letter and for the reviewers’comments concerning our manuscript entitled “The Genotype Distribution, Infection Stage and Drug Resistance Mutation Profile of Human Immunodeficiency Virus-1 among the Infected Blood Donors from Five Chinese Blood Centers, 2014-2017”. Those comments are all valuable and very helpful for revising and improving our paper, as well as the important guiding significance to our researches. We have studied comments carefully and have made correction which we hope meet with approval. Revised portion are marked in red in the paper. The main corrections in the paper and the responds to the reviewer’s comments are as flowing:

Responds to the reviewer’s comments:

Reviewer #2:

1.Response to comment: This article needs to provide GenBank accession number of 356 obtained sequences.

Response: We are very sorry for our negligence of  the Genbank Accession Numbers of 356 obtained sequences. We have provided GenBank accession number of 356 obtained sequences in line 129-131 of the manuscript.

2.Response to comment: Line 62: “CRF_08BC” should be “CRF08_BC”.

Response: We are very sorry for our incorrect writing the CRF08_BC. We have corrected it on line 62 of the manuscript.

3.Response to comment: Line 113-114: “JP.AB845344_K231, JP.AB845345_K320; CRF62_BC: KC870035.1, KC870034.1, KC870037.1” should be “AB845344, AB845345; CRF62_BC: KC870035, KC870034, KC870037”.

Response: This problem was caused by our carelessness and has been rechecked and corrected between lines 112 and 113.

4.Response to comment: Table 1: The sample size of CRF59_01B among five blood centers should be in regular font instead of bold.

Response: We are very sorry for our incorrect writing and we have corrected it.

5.Response to comment: Table 2: the total number of subtype B for recent infection should be “4”; the total number of subtype CRF01_AE for long-term infection should be “54”.

Response: We are very sorry for this small mistake, and the manuscript has been checked and revised over and over again.

6. Response to comment: The sentence of line 201 should be further improved.

Response: We have further polished this sentence. As the reviewer said, the language really needs to be further strengthened, and we will make more efforts in this regard.

7. Response to comment: Line 218-220: The sentence (Finding substantial cases of these four CRFs) seems not accurate because the numbers of CRF55_01B, CRF59_01B and CRF68_01B were 15, 1, and 1, respectively. In addition, “four CRFs” should be “three CRFs”.

Response: As the reviewer said, this sentence is indeed inaccurate, and we have revised it.

8. Response to comment: Line 228: “12.1% (12.1%, 43/356)” should be “12.1% (43/356)”.

Response: We have made correction according to the Reviewer’s comments on line 231.

9. Response to comment: Table 3: “250” should be “251”. “312” should be “313”. “355” should be “356”. The “P value” should be re-calculated.

Response: Thank you very much for the comment given by the reviewer. In the S1 Excel. China REDSIII HIV testing summary provided by us, the sample MY-12-003108 was not tested the infection stage, so this sample was excluded from the calculation.

10. Response to comment: The sentence of line 253-256 should be further improved.

Response:we are very grateful to the reviewer for his opinion on us, and we have examined the problems of English grammar carefully .

11. Response to comment:  Line 319: “A” should be “a”.

 Response: This problem was caused by our carelessness and has been rechecked and corrected at line 322.

12. Response to comment: The format of references 8, 53, 54, 55 and 56 should be checked.

Response: We have made correction according to the Reviewer’s comments on this question.

We have tried our best to improve the manuscript and made some changes to it. These changes will not affect the content and framework of the paper.We sincerely thank the editors / reviewers for their enthusiastic work and hope that the revision of this article will be recognized. Thank you again for your comments and suggestions.
